# Reconciling the statistics of spectral reflectance and colour

**DOI:** 10.1371/journal.pone.0223069

**Published:** 2019-11-08

**Authors:** Lewis D. Griffin

**Affiliations:** Computer Science, UCL, London, United Kingdom; University of Sussex, UNITED KINGDOM

## Abstract

The spectral reflectance function of a surface specifies the fraction of the illumination reflected by it at each wavelength. Jointly with the illumination spectral density, this function determines the apparent colour of the surface. Models for the distribution of spectral reflectance functions in the natural environment are considered. The realism of the models is assessed in terms of the individual reflectance functions they generate, and in terms of the overall distribution of colours which they give rise to. Both realism assessments are made in comparison to empirical datasets. Previously described models (PCA- and fourier-based) of reflectance function statistics are evaluated, as are improved versions; and also a novel model, which synthesizes reflectance functions as a sum of sigmoid functions. Key model features for realism are identified. The new sigmoid-sum model is shown to be the most realistic, generating reflectance functions that are hard to distinguish from real ones, and accounting for the majority of colours found in natural images with the exception of an abundance of vegetation green and sky blue.

## Introduction

A viewer sees an illuminated surface—the light arriving at their eye being determined by the intensity of the illuminant and the reflectance of the surface, both as a function of wavelength—and declares it ‘green’. The causal sequence underlying this threads through Popper’s three levels of reality [1]: world I, matter and sensation; world II, perception and cognition; world III, language and culture. Theories that link Popper’s worlds, are an important goal of science [2–4]; and colour presents a ‘goldilocks’ case study for this: not too simple, like temperature; nor too complex, like shape [5, 6].

There is more than one way to link Popper’s levels. Most prized are *mechanistic* models linking adjacent levels, and much has been achieved in colour science: electromagnetic models describe the interaction of the illumination with the scene [7]; optical ones, the formation of the retinal image [8]; electro-physiological, the retinal cone transduction of light into a nerve impulse [9–11]); and neuronal, the re-coding of sensory responses into opponent channel representations [12]. When levels are skipped, mechanistic models are not feasible, but accurate *predictive* models may still be; for example, colour naming atlases [13, 14] that link sensory responses to language. Sketchier yet, *consistency* between distant levels can be shown, for example between the cognitive similarity structure of the Basic Colours Terms [15] and the spectral sensitivities of retinal cones [16].

This study aims for a consistency linkage–to establish *a generative model of spectral reflectances functions*. We will evaluate the fidelity of the model in two ways -

- *that its reflectance functions should look like natural ones*, *and*
- *that the colours of those reflectances when illuminated should be distributed like those in the natural environment* -

using machine learning and statistical methods to quantify and to compare rival models. As well as contributing to the scaffold of colour science that links across Popper’s levels, such a model may be useful for other studies. For example in understanding colour constancy, in which illumination and sensation vary, while reflectance and perception are stable [17]; or in determining optimal gamut transformations between devices [18].

The relationship between the statistics of natural reflectance and natural colour has previously been studied using Principal Components [19, 20] and Fourier Analyses [21]. PCA methods were used to synthesize reflectance functions as the weighted addition of a small (~6) number of components. The realism of these functions was assessed only informally but, ignoring that a few percent of such generated functions will have reflectance values outside the valid range [0,1], was clearly good. The distribution of colours of these reflectances was not assessed for naturalness. While PCA is conceptually simple, the model is not compact, requiring ~200 values to be specified (mean + 6 components × number of wavelengths sampled).

The same study [19] that considered PCA for analyzing spectral reflectance also proposed that natural reflectances were effectively band-limited in the frequency domain. A fuller analysis [19, 21] of the frequency domain characteristics of reflectance functions showed that they were better considered to have a power-law form decrease of energy with frequency, rather than a sharp cut-off. This led to a very compact model of spectral reflectance functions with only a few parameters (mean reflectance, spectral power, power-law exponent fall-off). This work only informally assessed the realism of the generated functions and their distribution of colours. As we will show later, with some adjustments they can both be very good.

In this paper we will introduce methods objectively to quantify the realism of models of spectral reflectance, in terms of individual functions and in terms of the distribution of their colours. We will apply these methods to previous PCA and fourier-based models, to understand their weaknesses, and will propose new models that are more realistic. We will achieve a model that is simple and realistic, which we hope will find use in future research.

The paper is organized as follows. In section 2 we present *datasets* of spectral reflectance and natural colour. In section 3 we describe the *methods* we use to assess the realism of a model of spectral reflectance. In 4 we describe *formation* models for the colour-from-reflectance process. Using these formation models we show that the reflectance and colour datasets are not very consistent. In 5 we describe previous models (*PCA*, *fourier*) of the distribution of spectral reflectance, and show that neither produce both realistic spectra and colours. In 6 we extend these previous models (*PCA+*, *fourier+*) which improves their realism, and we present a new *sigmoid-sum* model which is the most realistic. In 7 we discuss the key features needed for models to be spectrally and colour realistic, and the realism of the *sigmoid-sum* model. In 8 we summarize and conclude.

## Data

We describe the datasets, collected by others, of natural spectral reflectance functions and colours that we use to evaluate realism.

### 2.1 Spectral reflectance functions

A spectral reflectance function maps from the visible spectral interval Λ≔[380,720] nm to the reflectance range [0,1]. It specifies, as a function of the incident illumination wavelength, the energy of the light reflected by a surface as fraction of that reflected by a white lambertian surface.

**Fig 1:**
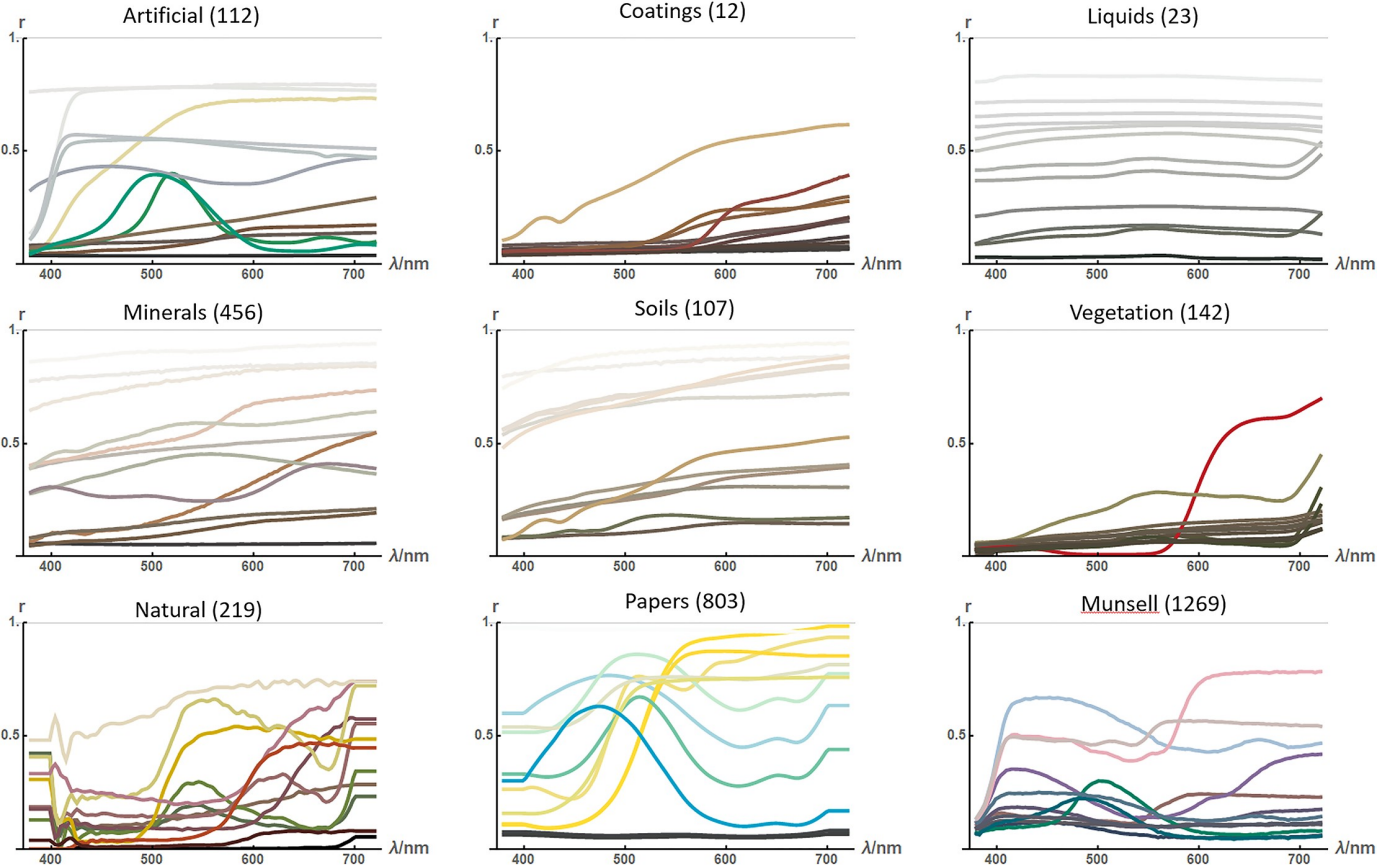
Shows twelve random examples from each of nine collections of spectral reflectances functions. Plot labels indicate the theme and size of each collection. Reflectance functions are plotted in the colour they would appear under daylight (D65) illumination.

For statistics on spectral reflectance we merge nine *collections* from two sources [22, 23] (Fig 1). These collections vary in their wavelength range and sampling, but we extrapolate and interpolate them into a common scheme. Where extrapolation has been used (for example from 400nm to 380nm for the ‘natural’ collection) we consider this harmless for further analysis.

The collections vary in the type and number of materials measured. We presume that each collection was assembled with an eye to diversity, rather than as a random sample of a natural population. Overall, we consider the total dataset as informative of the appearance of reflectance functions (smoothness etc.) but not as a reliable model of their natural distribution.

We make a few informal observations on the functions shown in Fig 1.

- Increasing segments are more common than decreasing.
- Most functions have fewer than five extrema. The ‘natural’ collection has additional high-frequency structure presumed to be measurement noise.
- The functions can be parsed as mostly made of plateaus and slopes, occasionally sigmoid steps, less frequently humps, and rarely dips.

The collections vary in their wavelength sampling. For consistency all functions were re-sampled (cubic interpolation) at 128 wavelengths equally-spaced across Λ. When appropriate each collection is weighted equally, nullifying the variation in their size.

We have studied the frequency power spectra of the functions, revisiting the analysis performed in [21]. Numerical frequency analysis assumes periodic signals, which is clearly not the case here. If unaddressed, the resulting spectra are strongly influenced by the wraparound discontinuity. We deal with this by removing a linear trend from each reflectance and then apodizing it with a Gaussian window (*μ* = 550*nm*, *σ* = 50*nm*). The removal step is computed so that the apodized function is free of linear trend. After detrending and apodization we use an FFT to compute frequency power spectra, remove the three lowest frequency terms (because corrupted by the detrending), normalize each power spectra to unit sum, average across a collection, and then square root to obtain an overall frequency amplitude spectrum.

**Fig 2:**
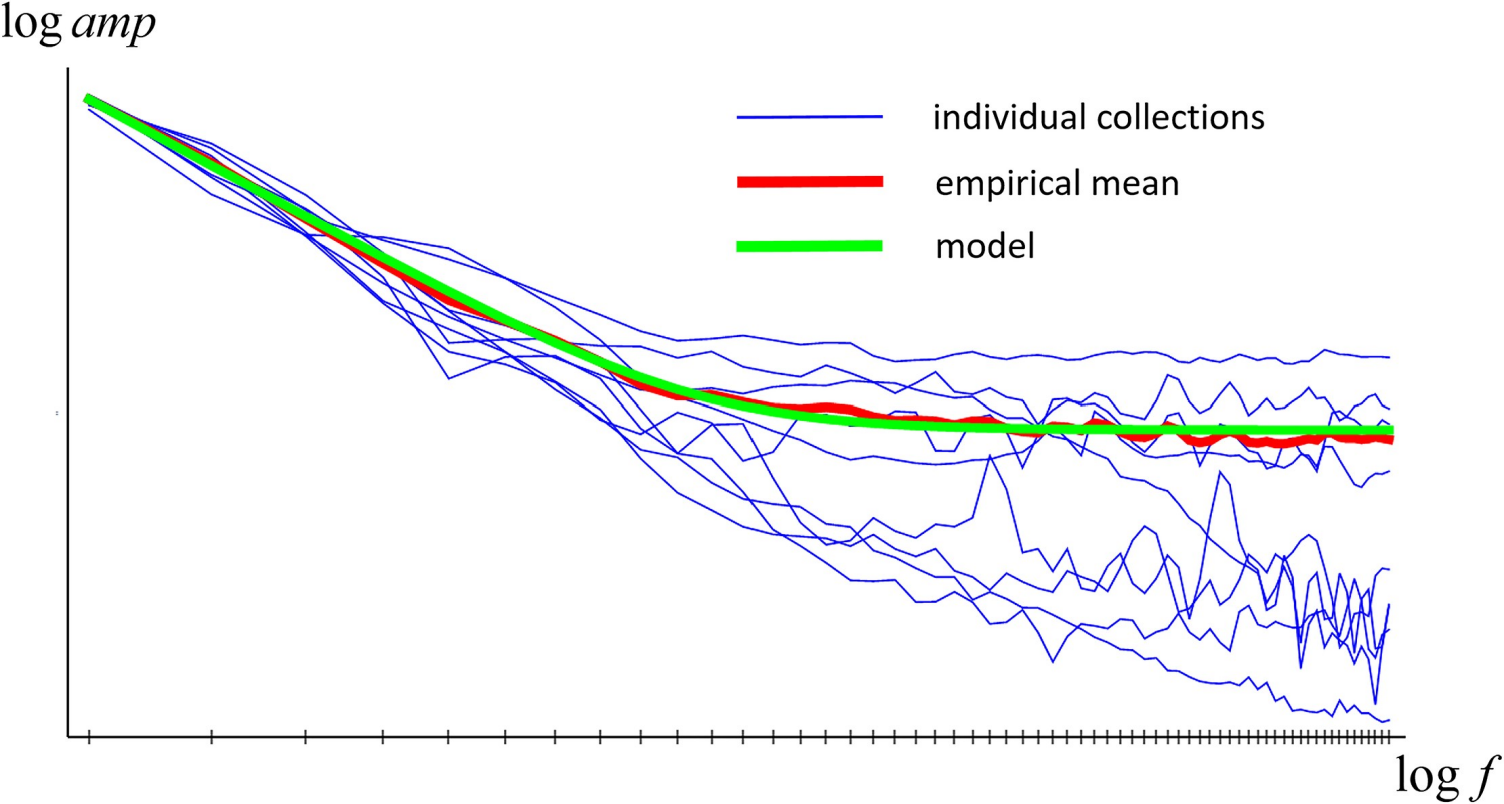
Frequency amplitude spectra for individual collections (blue), averaged over collections (red), and modelled (green).

In Fig 2 we plot the results as log amplitude vs. log frequency. As the figure shows, on such plots the amplitudes have a decreasing linear form for low to medium frequencies, and then a constant value for higher frequencies. The linear part arises from a power law form dependency of amplitude on frequency (i.e. *a*∝*f*^−*γ*^), while the constant part arises from measurement noise. We have fitted a model of this to the data (see Fig 2) which recovered a power law coefficient of *γ* = 2.3. Our results thus confirm the power-law frequency amplitude model proposed in [21], but there the estimate of *γ* = 1.8 was lower–larger coefficients corresponding to smoother functions.

### 2.2 Colour

For data on the colours present in the natural environment we use the RGB values of a large collection of images. This is a convenient alternative to the use of spectroradiometers [24] or hyper-spectral imaging systems [25]. While those methods do allow accurate prediction of the colour sensations provoked by scenes, their technical complexity makes it difficult to assemble large datasets. In contrast, RGB digital cameras are a simple to use, widely-available technology from which large datasets already exist. The detraction of using digital cameras is that the accuracy of colour estimation is limited by the agreement between the null space of the camera and cone spectral sensitivities, but increasingly the compromise of RGB cameras has been accepted for statistical studies [26, 27].

**Fig 3:**
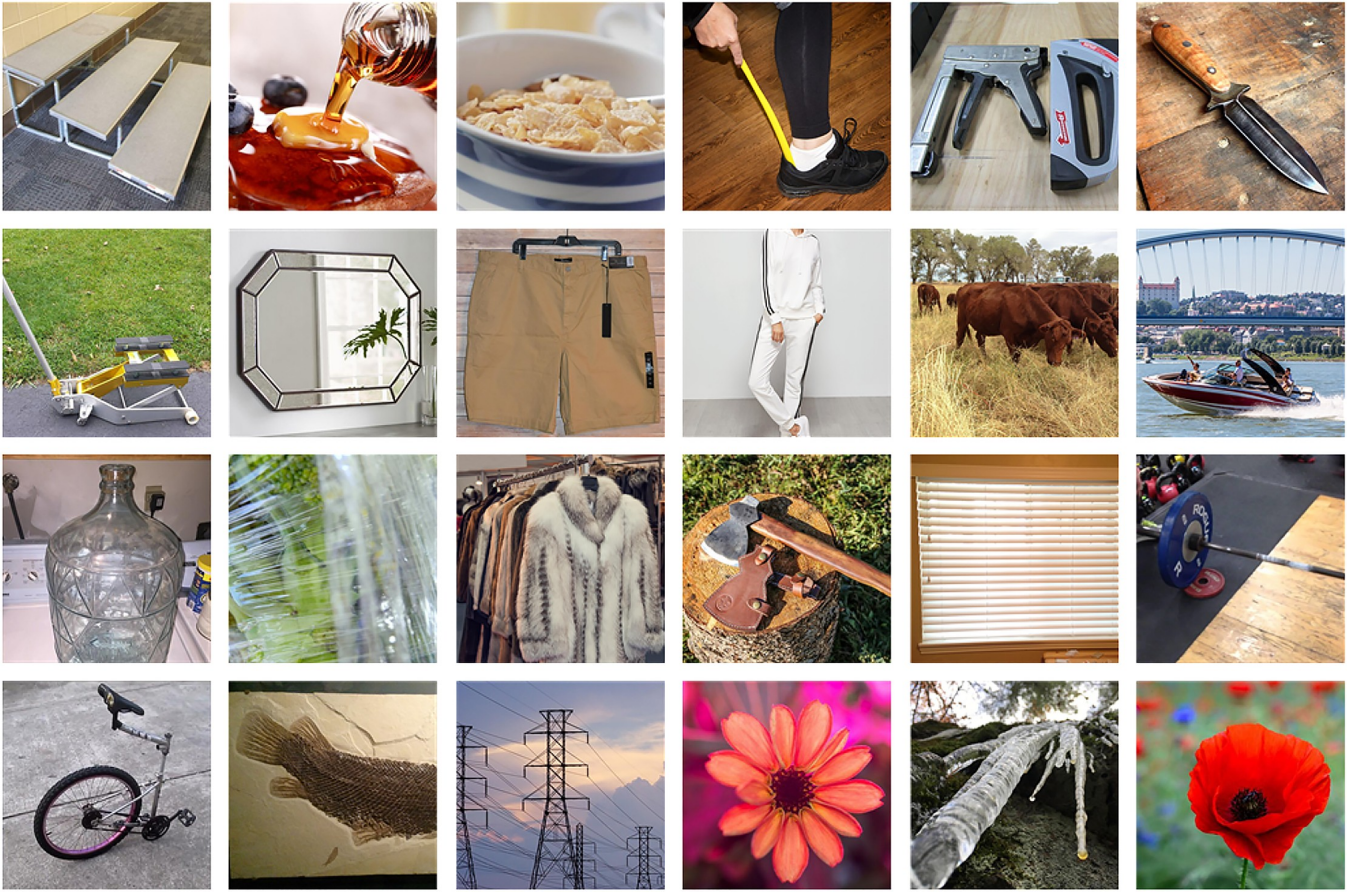
Example images from the THINGS dataset.

Any image database that we could use is open to criticism as to its ‘naturalness’, since the term is vague and, to the extent that it is not, is surely variable with time and place. We acknowledge this but choose the THINGS dataset [28] which has been assembled for research in psychology, neuroscience and computer science. This dataset consists of 26,107 images organized into 1,854 common object categories according to the main object present within them. Each image is 800×800 pixels and has been manually cropped from a larger source image. The source images were collected by internet search for each category, and selected from the results according to quality, variation and absence of artefacts such as text or borders. Examples are shown in Fig 3.

**Fig 4:**
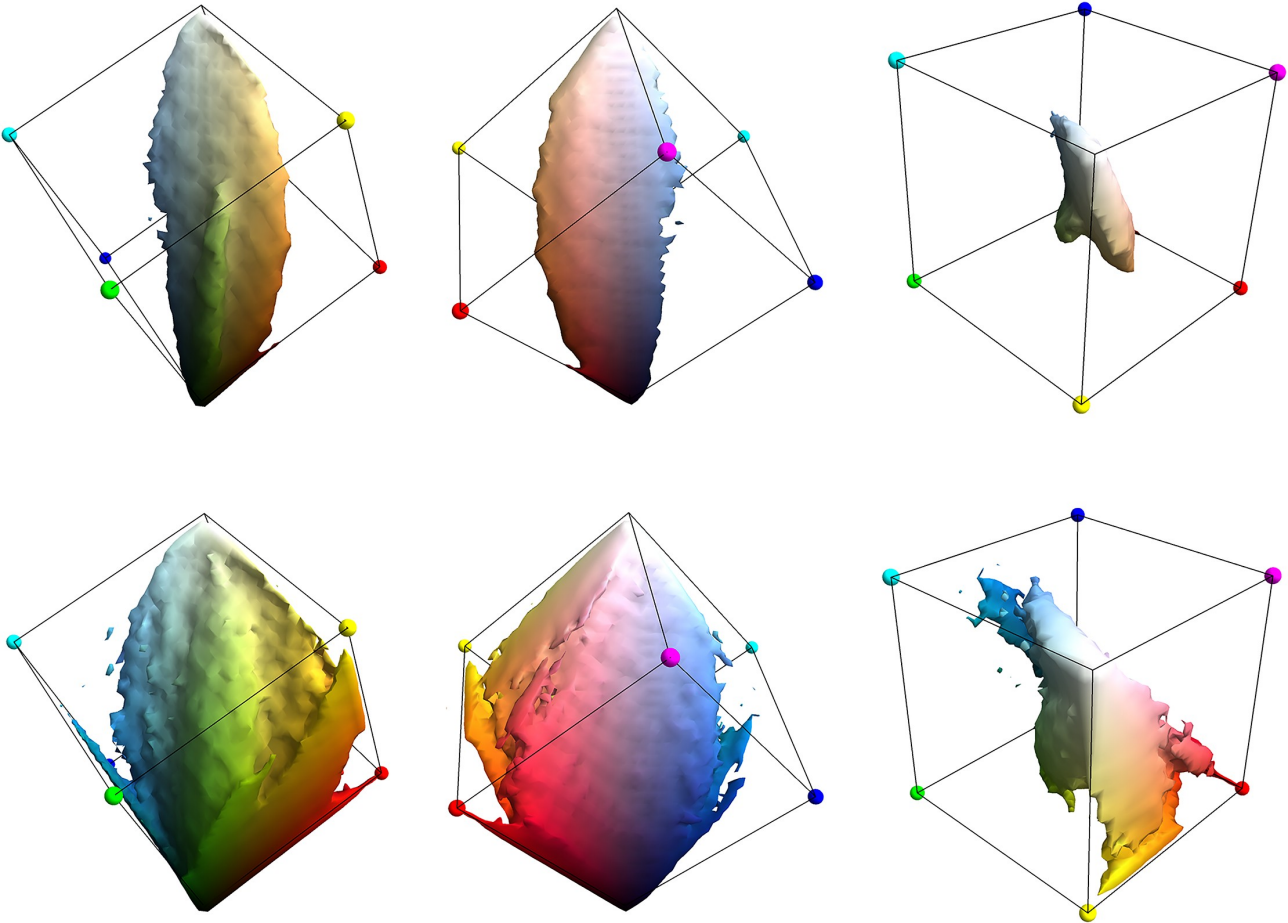
The mean RGB histogram of the THINGS dataset. All views show iso-density surfaces of the histogram, coloured according to the corresponding RGB Value. The top row shows views of the iso-density surface that encloses 80% of the mass of the histogram, the bottom row enclosing 96%.

Although the variation across images in their RGB content is a topic of great interest, here we simply compute the overall histogram for the entire dataset (equivalently the arithmetic mean of the per-image histograms). The image data is all 8-bit depth in each channel, but to ease later computations we compute the histogram at 5-bit depth, hence with 2^5^×2^5^×2^5^≈30K bins. Since even 2-bits per channel is sufficient roughly to segregate the 11 Basic Colour categories [29] we are confident that this is adequate resolution. This *mean histogram* is visualized in Fig 4, with observations:

- There is a high-density core around the achromatic axis [27] containing 80% of the colours present in the images (Fig 4, top row).
- The core has roughly the shape of a thick, asymmetric, elliptic leaf with the central vein running along the achromatic axis. The plane of the leaf extends in the orange-blue direction, further into orange than blue. The thickness of the leaf is in the purple-green direction [27].
- Against the plane of the leaf, lies an additional ‘worm’ of higher density extending from ‘leaf green’ towards black, presumably due to the commonality of vegetation in the environment; and along one edge, a ‘fringe’ extending from ‘sky blue’ towards white, presumably due to sky.
- The tails of the histogram (Fig 4, bottom row) are not a simple extension of the core.

## Assessing realism

Our aim is to develop a model which produces individually realistic spectral reflectance functions, with a realistic distribution of colours. We describe how we quantify realism.

### 3.1 Spectral functions

We assess the realism of a model of reflectance functions by how well they can be distinguished from empirical functions. We measure this using machine learning methods. Specifically, we train a classifier to distinguish between empirical and synthetic functions, and then test how well it performs on a test set. If the test classification is correct at chance (50%) levels we consider the synthetic functions to be realistic, but if performance is higher the classifier must have discovered distinguishing features so we consider them as unrealistic.

We use both random forest [30] and elastic-net regularized logistic regression [31] for classification, reporting the better performing of the two. We *train* a classifier using: as examples of empirical reflectances, 12 randomly selected from each of all but one of the collections (thus 96 = 12×(9–1) examples); and, as examples of synthetic reflectances, 96 functions randomly generated by the model. The size of each training set (only 12 reflectances per collection) is determined by the smallest collection, and the desire to represent the collections equally, to best capture the cross-collection variation.

We *test* the classifier using 12 random reflectances from the left-out collection and 12 randomly generated synthetic reflectances. We score the classifier by the rate at which it gives a higher confidence for an empirical test reflectance than for a synthetic (equivalent to the area under the ROC curve). We repeat the assessment hundreds of times, leaving out different collections, and take the overall average score as the final measure. We perform two modes of assessment. One as described, and one *colour-balanced* in which, in both train and test sets, each model-generated reflectance is colour-matched to an empirical reflectance. Colour-matching is achieved by creating model reflectances until one of the correct colour is generated. The colour-balanced mode prevents the classifier using the diversity of reflectance functions as a cue, forcing it to attend only to their individual form.

### 3.2 Colour

We assess the colour realism of a model of reflectance functions by using a formation model (section 3) to compute the image colours of the reflectances; compiling those colours into an RGB histogram; and comparing that histogram to the mean image histogram. We use three well-known measures to compare RGB histograms.

The simplest measure is the Intersection Distance (ID) which focusses on how much mass the two histograms have in common. It measures the percentage of histogram density that is specific to one of the histograms; the lower it is, the more similar the histograms. ID focusses on the size of histogram bin count mismatches, ignoring colour distance.

A measure that does considers colour distance is the Earthmover’s Distance (EMD) which measures the mean distance that mass must be transported optimally to transform one histogram into the other. We express the EMD relative to the side length of the RGB cube.

The third measure is Jensen-Shannon Divergence (JSD) which tells how much information (measured in bits) is on average gained about which was the originating histogram given a single colour picked randomly from one or other of the histograms compared. Like ID, JSD ignores colour distance, but unlike ID it is particularly sensitive to bin count mismatches in the tails of the histograms.

**Fig 5:**
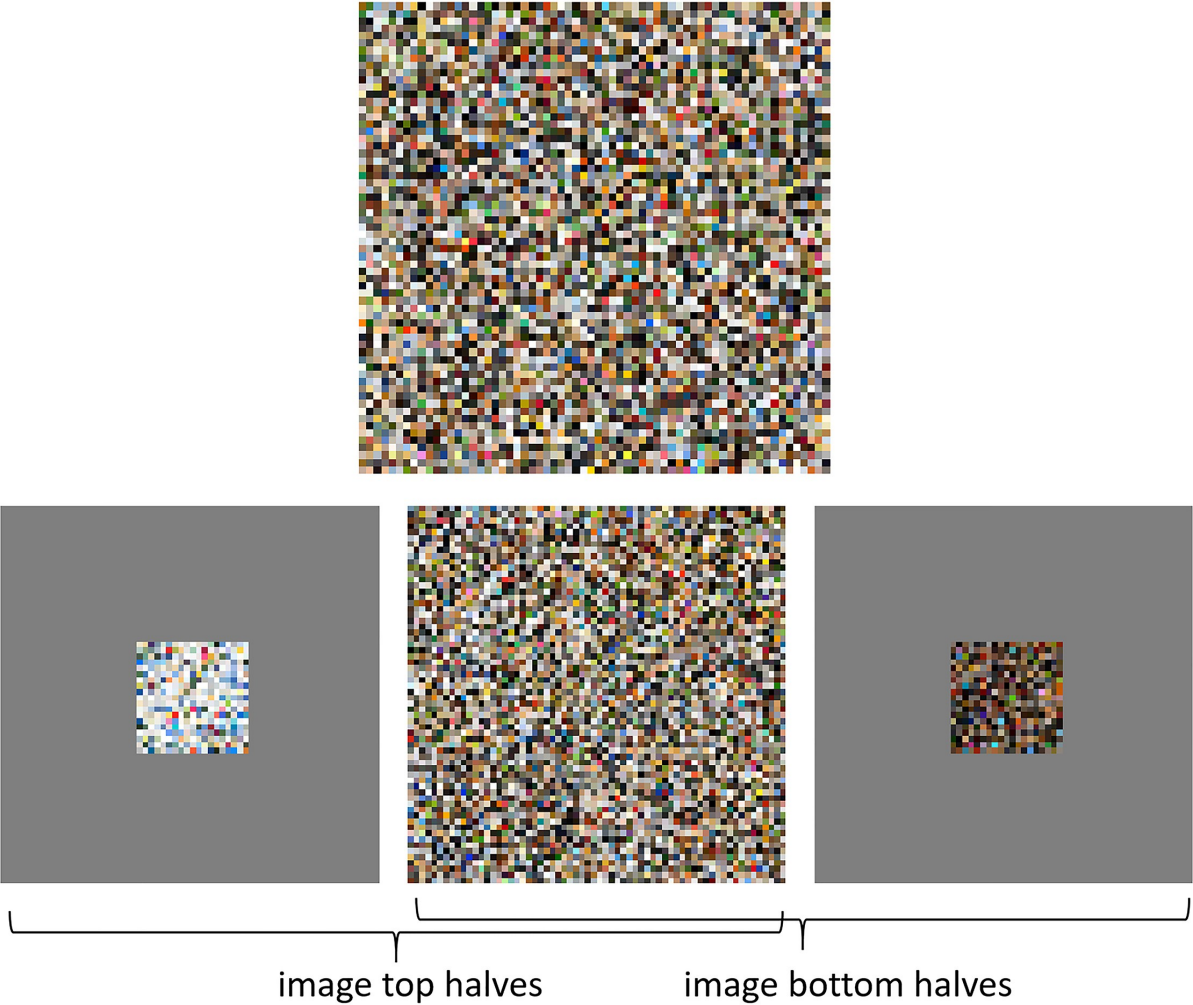
Visualizes RGB histograms using swatches, which are random samples of colour from the density. The top swatch is for the mean image histogram. The bottom row compares the top (*T*) and bottom (B) half-image histograms. The central swatch is for the intersection histogram (min(*T*,*B*)); the left and right swatches for the excesses (*clip*_+_(*T*−*B*) and *clip*_+_(*B*−*T*)). The swatch sizes reflect the density mass in the intersection and excess histograms, so the left and middle swatches combined are a swatch for the top-half histogram. The ratio of sizes is 0.08:0.92 so the *intersection distance* (ID) between the half histograms is 8%.

To further introduce the measures we compute them for an example pair of histograms, one derived from the top half of the THINGS image dataset, and the other from the bottom half. These ‘half histograms’ are visualized in Fig 5 which also introduces *swatches*, an alternative to iso-density visualization. The figure shows that the top histogram (*T*) has extra pale blue and white colours, and the bottom (*B*) extra black and brown. This is due, no doubt (Fig 3), to more frequent sky/clouds above and shadows/earth below (see also [27]). The distance measures between these half histograms are: ID = 8%, EMD = 3.5% and JSD = 0.01. We will focus on ID in the remainder because of its easy interpretation.

## Colour-from-reflectance formation models

Our aim is to assess the consistency between reflectance and colour natural statistics. To do that we predict the distribution of colours that will arise from a sample or distribution of reflectances, and compare that to the distribution of colours observed in images. To predict colours from reflectances we require *formation* models. We consider two: a *simple* uniform-illumination model and a more complex *Ostwald* shading+specularity one.

The simple formation model assumes uniform average daylight illumination [32] specified as a spectral energy function $D_{65}:\Lambda \to {\mathbb{R}}^{+}$; Lambertian surfaces; the CIE 2012 2° xyz colour matching functions $\vec{m}\left(\lambda \right)≔{\left(x\left(\lambda \right)\;y\left(\lambda \right)\;z\left(\lambda \right)\right)}^{T}$ (derived from [33]); and the standard conversion matrix $M≔\left(\begin{array}{ccc} -3.2406 & -1.5372 & -0.4986\\ -0.9689 & 1.8758 & 0.0415\\ 0.0557 & -0.204 & 1.057 \end{array}\right)$, non-linearity and clipping function $\eta \left(z\right)≔clip_{\left[0,1\right]}\left[\{\begin{array}{cc} 12.92z & z\leq 0.0031308\\ 1.055z^{1/2.4}-0.055 & z>0.0031308 \end{array}\right]$ to convert to sRGB [34]. The model produces a colour $\vec{c}≔{\left(r\;g\;b\right)}^{T}$ from a reflectance function *ρ*:Λ→[0,1] according to:

$$\rho \to \vec{c}=\eta \left(M\left(\int_{\lambda \in \Lambda }\vec{m}\left(\lambda \right).D_{65}\left(\lambda \right).\rho \left(\lambda \right)\right)/\left(\int_{\lambda \in \Lambda }y\left(\lambda \right).D_{65}\left(\lambda \right)\right)\right)$$

**Fig 6:**
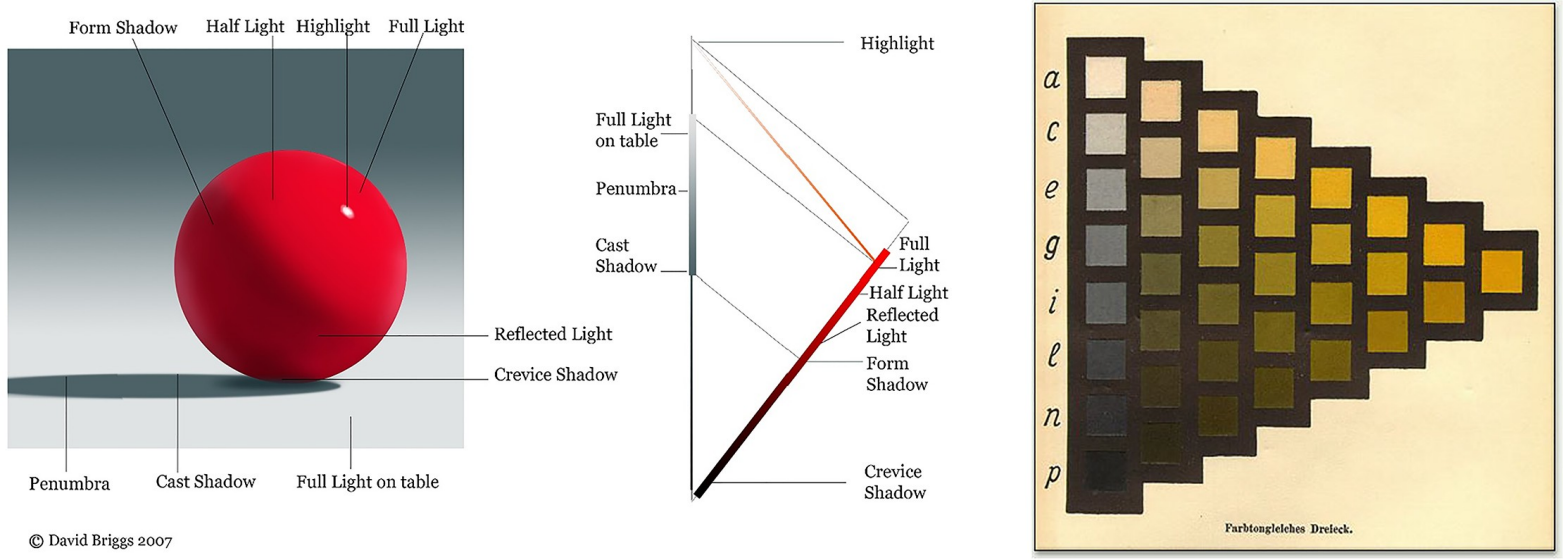
The left and centre panels illustrate the colours arising from shading and specularity in the case of an object with homogeneous spectral reflectance. At right is shown an example page from the Ostwald Atlas, which can be considered a chart of the colours that can arise in this way.

The simple formation model ignores shading (non-uniform illumination intensity) and highlights (specular reflection) which are ubiquitous in natural scenes (see Fig 3). Fig 6 illustrates how these processes effect colour: shading shifts colour towards black, and specularity towards white, neither process effecting hue [35].

The Ostwald formation model incorporates shading & specularity by making use of the Ostwald triangle representation [36] which organizes the possible *effective colours* of a homogeneous surface (Fig 6 right). The effective colours arise as partitive mixtures of the full colour, black and white–a partitive mixture being a weighted additive mixture, where the weights are positive with unit sum [37].

The Ostwald triangle can also be considered as organizing the *effective reflectances* of a surface. Let *ρ* be a surface reflectance function; $\vec{c},\vec{b},\vec{w}\in {\mathbb{R}}^{2}$ the vertices of the Ostwald triangle corresponding respectively to full illumination, shadow and specularity; and let 〈*o*_*c*_,*o*_*b*_,*o*_*w*_〉 be barycentric coordinates [38] (constrained by *o*_*c*_,*o*_*b*_,*o*_*w*_>0, *o*_*c*_+*o*_*b*_+*o*_*w*_ = 1) indexing a point $o_{c}\vec{c}+o_{b}\vec{b}+o_{w}\vec{w}$ in the triangle. The effective reflectance associated with that point is *ρ*_*eff*_≔*o*_*c*_*ρ*+*o*_*w*_.

**Fig 7:**
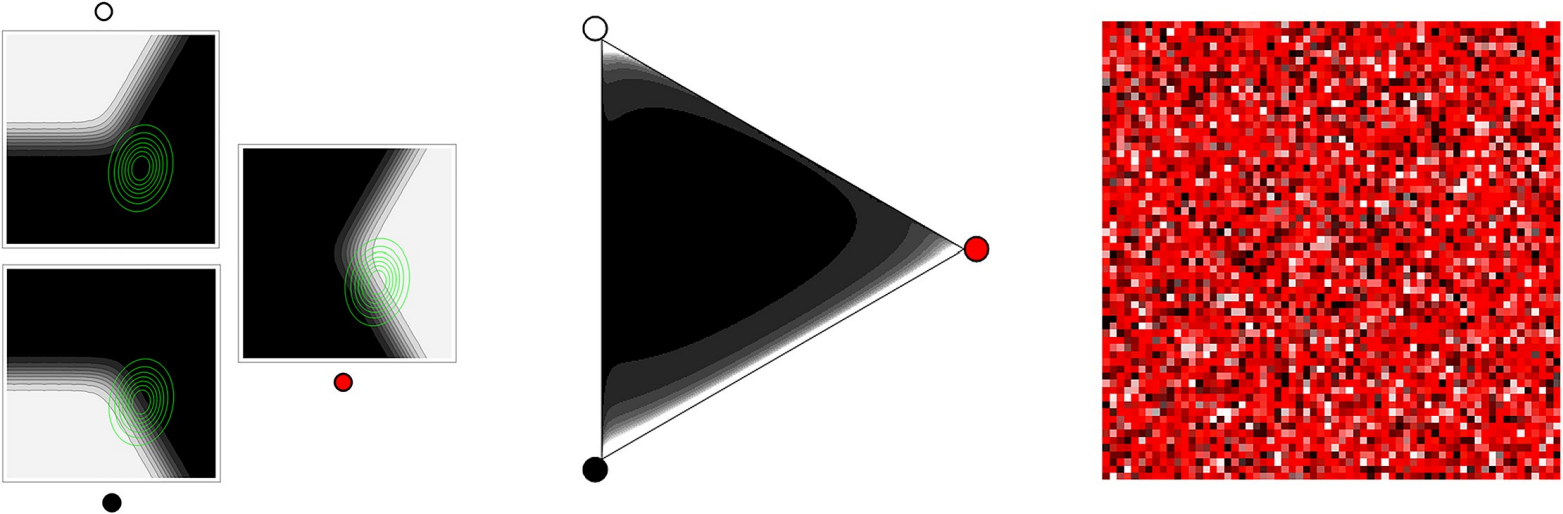
An example Ostwald distribution. Left: Ostwald triangle coordinates as functions of the latent domain; with an example bivariate normal distribution superimposed (green). Middle: the bivariate normal distribution transformed to the Ostwald triangle. Right: a swatch from the Ostwald triangle distribution.

We model the effects of shading and specularity in the colour-from-reflectance process using a distribution over the Ostwald triangle to capture how often different degrees of shading and/or specularity occur during the formation of an image. We represent that distribution using a bivariate normal distribution over a latent variable domain $\vec{l}\in {\mathbb{R}}^{2}$, which transforms to Ostwald coordinates according to $\vec{l}\to \langle o_{c},o_{b},o_{w}\rangle =\langle e^{\vec{l}\cdot \vec{c}},e^{\vec{l}\cdot \vec{b}},e^{\vec{l}\cdot \vec{w}}\rangle /\left(e^{\vec{l}\cdot \vec{c}}+e^{\vec{l}\cdot \vec{b}}+e^{\vec{l}\cdot \vec{w}}\right)$. Fig 7 (left) shows Ostwald coordinates as a function of the latent domain, with an example bivariate normal distribution superimposed (green). This distribution over the latent domain transforms to a distribution over the Ostwald triangle (Fig 7, centre). In the example shown most of the mass is along the shadow series from full illumination to black, while the remaining mass is near the white specularity corner of the triangle. Fig 7 (right) shows a swatch for that distribution. It corresponds roughly to the distribution of colours arising from the sphere in Fig 6 (left).

Using the simple and Ostwald formation models, we have computed the RGB histograms that result from the dataset of empirical reflectances. As previously noted, we sample equally from the nine collections that form the dataset, even though they have different numbers of members. The process is (i) randomly sample a reflectance from the dataset, (ii) compute its colour according to the formation model, (iii) accumulate a count in the corresponding cell of the RGB histogram, (iv) repeat 1M times. Step (ii) is deterministic when using the simple model, and stochastic when using the Ostwald model. In the latter case we generate a sample from the bivariate normal Ostwald distribution, and convert it to Ostwald triangle coordinates; from these coordinates and the sampled reflectance we compute an effective reflectance; then compute its colour as per the simple model.

Here, and whenever we use the Ostwald formation model, the five parameters of the bivariate normal distribution (Fig 7, left, green) have been tuned to minimize the ID between the resulting histogram and the mean histogram. Though the exact parameters vary, in all cases the optimized distribution is similar to the example shown in Fig 7.

**Fig 8:**
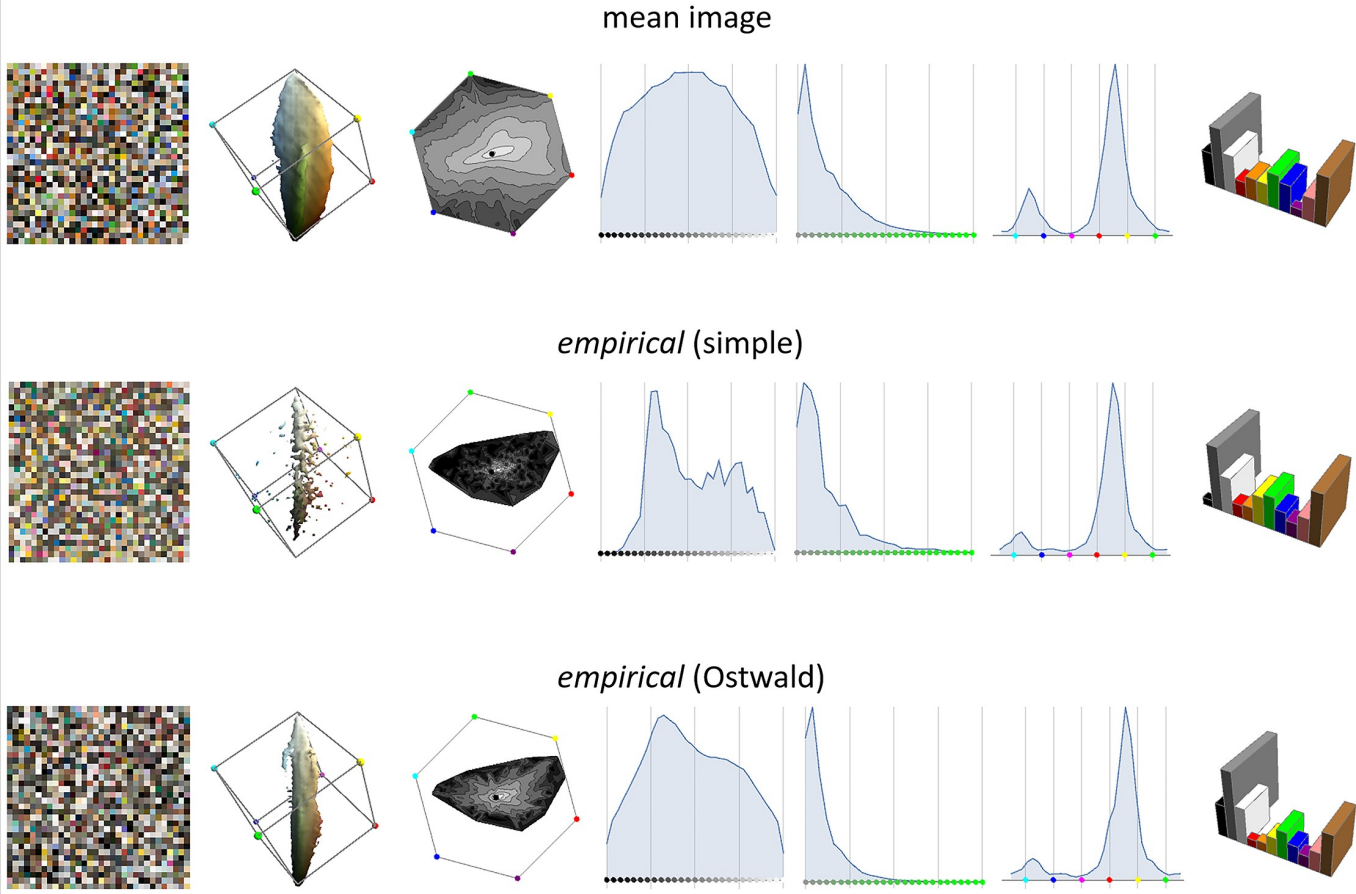
Each row shows a gallery of visualizations for an RGB histogram. Reading left-to-right these are: a swatch; the 80%-mass-containing iso-density surface; the hue×saturation marginal (grey-level indicating log-density); the lightness marginal; the saturation marginal; the hue marginal; the Basic Colour Category marginal.

Fig 8 shows the mean image histogram and the *empirical* histograms from the reflectance dataset when using the two formation models. For each histogram we use a gallery of visualizations that we will use throughout, with notes:

- the hue×saturation marginal is plotted with grey-level indicating log density, greatly exaggerating the higher saturation flanks, but allowing them to be seen;
- saturation marginals typically peak at low, but non-zero, saturation; even though the hue×saturation marginal density peaks at achromatic. This can happen because there are more low-saturation histogram bins than achromatic.
- The Basic Colour Category marginal is computed using a soft assignment of histogram bins to Basic Colours computed from a large corpus of colour naming data [39]. In detail, ~20K unconstrained colour naming responses to 600 RGB specified colours were collected in an online experiment from ~1K subjects. A gaussian-form model of the response rate for each produced colour named was fitted as a function of RGB coordinates. Response functions for variants of individual basic colour (e.g. ‘red’, ‘strong red’, etc.) were summed. Colour terms that involved multiple basis colours were allocated to the involved colours depending on the syntax of the term (e.g. for ‘orange-red’ 50% of the weight went to ‘orange’ and 50% to ‘red’, whereas for ‘reddish-orange 66% went to ‘orange’ and 33% to ‘red’). The combined basic colour response functions allowed a distribution of basic colour responses to be determined for any RGB value. For many RGB triples this was a sharp correspondence (e.g. 100% ‘red’) whereas for others it was fuzzy (e.g. 70% ‘red’, 20% ‘orange’, 10% ‘pink’). The fuzzy assignment of RGB to basic colours has been included in the downloadable material for this publication. The area of the square indicates the mass in a bin.

Comparing the histograms in Fig 8 we observe:

- With the simple formation model the *empirical* histogram is too spikey as the number of reflectance functions is small compared to the number of histogram cells; this is improved by using the Ostwald formation model.
- With the simple formation model the *empirical* histogram has much fewer very dark colours (and to a lesser extent) very light colours than the mean histogram; this is improved by using the Ostwald formation model.
- The saturation marginal of the *empirical* histogram is a good match to the mean histogram with the simple formation model, but becomes too low in saturation when using Ostwald shading.
- The hue marginals are all in fairly good agreement, except purples are rarer and blues more common in the mean histogram.
- The Basic Colour marginals are in fairly good agreement, except for achromatic colours with the simple formation model.

In Table 1 we give distances between the mean histogram and the empirical histograms computed using the two formation models. As context, recall that the distances between the top- and bottom-half histograms were JSD = 0.01, EMD = 3.5% and ID = 8%. The figures in the table show that distances are much smaller with the Ostwald than the simple formation model. These reduced distances, together with the fact that the tuned Ostwald distribution (Fig 7, middle and right) agrees with expectation, validate the use of the Ostwald formation model as a necessary improvement over the uniform-illumination model.

**Table 1:** Distances between the mean image histogram and empirical histograms. JSD = Jensen-Shannon Divergence; EMD = Earthmover’s Distance; ID = Intersection Distance. The ‘+5’ is a reminder of the number of parameters tuned to minimize the ID.

formation model					
uniform-illumination			Ostwald (+5)		
JSD	EMD	ID	JSD	EMD	ID
0.53	4.2%	69%	0.16	2.1%	36%

## Previous reflectance models

**Fig 9:**
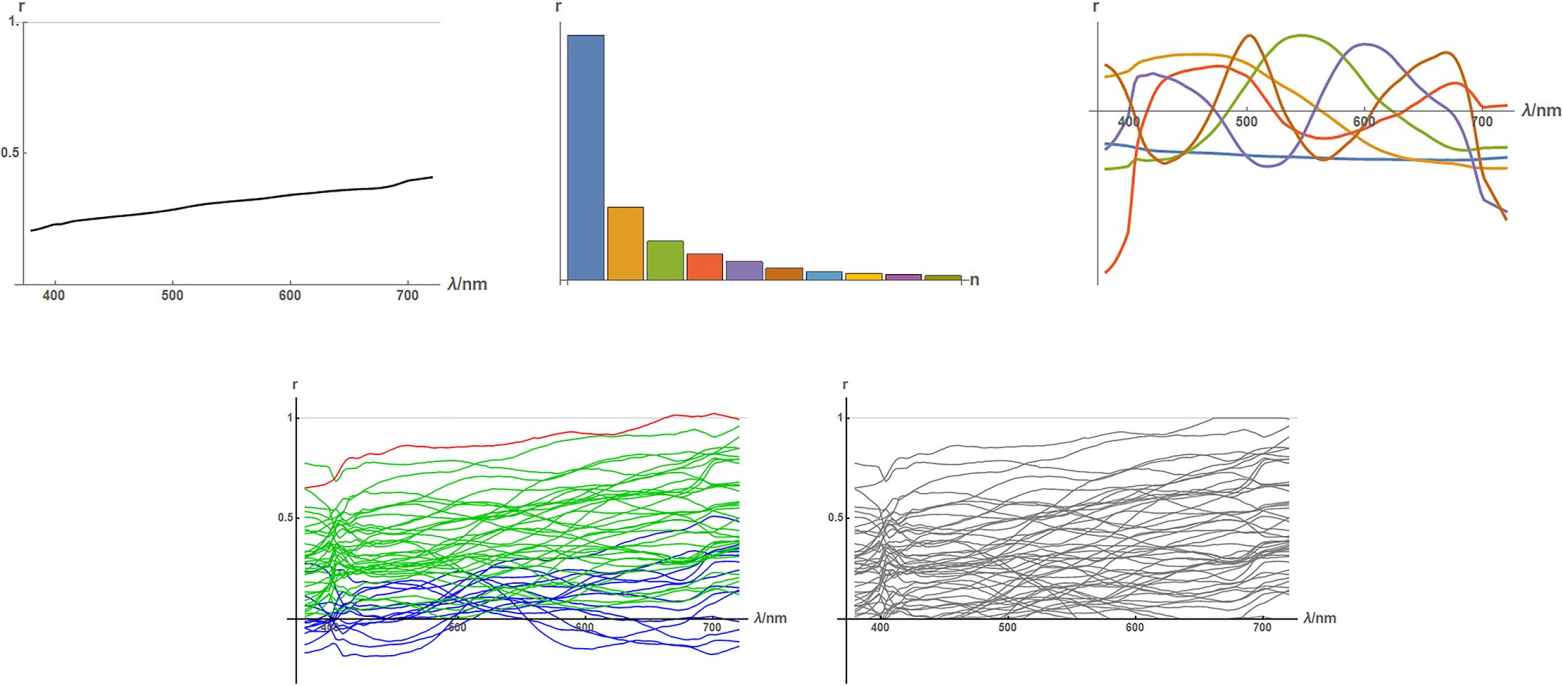
The *PCA* model of spectral reflectance. Top-left: the mean reflectance function. Top-middle: standard deviations of principal components. Top-right: first six principal components, colouring corresponds to the top-middle panel. Bottom-left: reflectances generated by the PCA model; a fraction (red and blue) have values outside the legitimate [0,1] range. Bottom-right: generated reflectances after clipping to [0,1].

Instead of using a dataset of reflectance functions directly as a model of the distribution of natural reflectances, one can generalize from it in the form of a generative model. Two such models have previously been described. The best-known of these (*PCA*) uses Principal Components Analysis [40] to construct a high-dimensional normal approximation [19, 20, 41]. Fig 9 shows such a model built using our empirical dataset (giving equal weight to each collection). A few things are of note:

- The mean reflectance has a positive slope.
- There is a power-law like decay in the magnitude of components.
- Components are roughly sinusoidal with frequency increasing with rank.
- A fraction of generated components have values outside the legitimate [0,1] range for reflectance, but this can be remedied by clipping.

The second previous model (*fourier*) [21] builds on the regularities observed in PCA: specifically that components are sinusoidal of increasing frequency, and their amplitudes follow a simple pattern. Like *PCA* it models the distribution of reflectance functions as a high-dimensional normal distribution but, rather than data-determined components, it uses sinusoids of increasing frequency (integer number of cycles over Λ), amplitude which decays in a power law fashion with frequency, and random phase. The *fourier* model is very compact–all that is needed to specify it is (i) the value of the DC component, (ii) the overall amplitude of the fourier components, (iii) the exponent of the power law decay.

**Fig 10:**
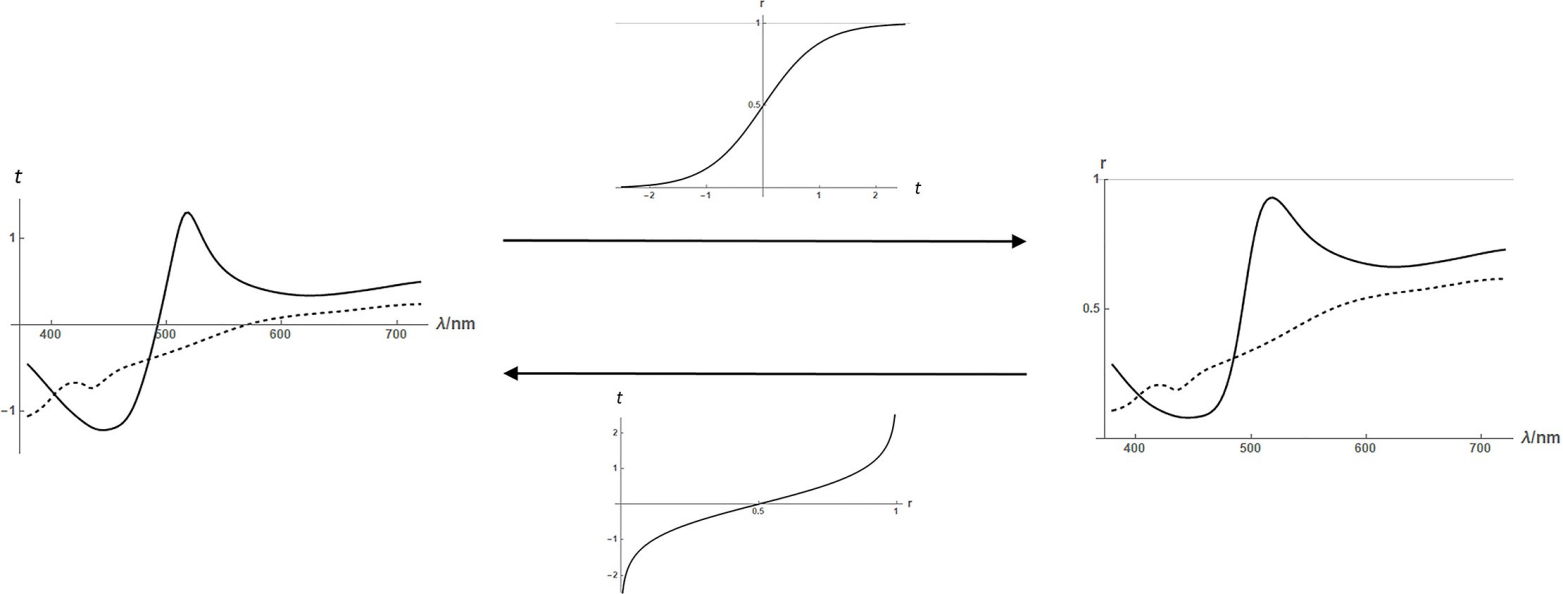
The latent variable (*t*) representation of reflectance (*r*). Functions from wavelength to *t* (left) can be converted to functions from wavelength to *r* (right) using the sigmoid transfer function (middle, top), or back the other way using its inverse (middle, bottom).

In the original description of the *fourier* model [21] it is argued that it is advantageous to represent reflectance *r*∈[0,1] using an unbounded latent variable t∈ℝ related by *t*→*r*≔(1+tanh*t*)/2 and *r*→*t* = tanh^−1^(2*r*−1) (see Fig 10). This removes the need to clip out-of-range functions that occurs otherwise (Fig 9, bottom), and more significantly allows better representation of functions with extreme reflectance values. We have implemented the *fourier* model from [21] using the parameter values there specified: a DC value of -1.0, a stochastic standard deviation of 1.2 per wavelength (both values in latent domain units) and a power law exponent of 1.8.

**Fig 11:**
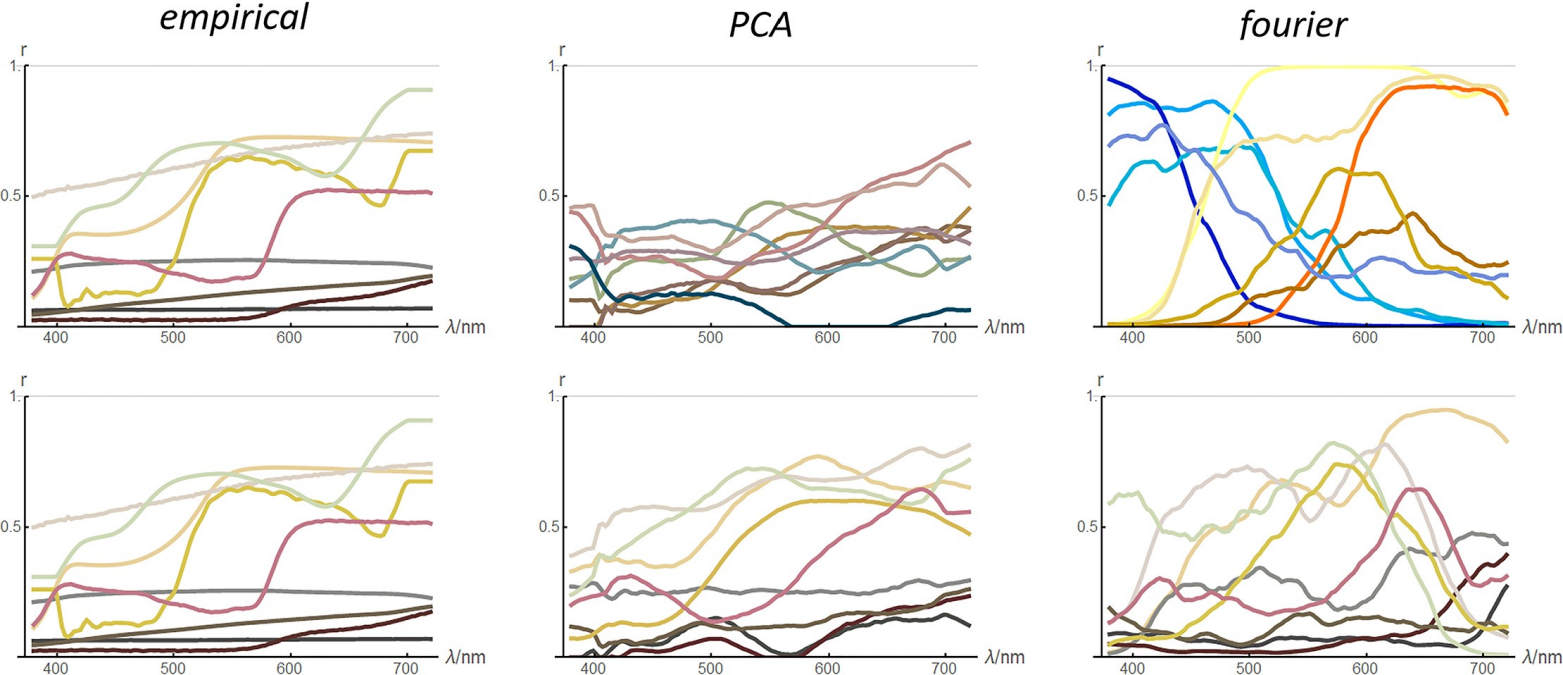
Comparison of empirical (left) and modelled (middle and right) reflectance functions. All functions are plotted in their colour under the uniform-illumination formation model. Left column: one function randomly chosen from each of the nine collections (same in top and bottom panels). Top row: unconstrained model reflectance functions. Bottom row: colour-matched model reflectance functions.

In Fig 11 reflectance functions generated by the *PCA* and *fourier* models are compared to *empirical* reflectances. The top row of the figure shows random model functions, while the bottom row shows random model functions chosen to colour-match the empirical reflectances. Visualizing the two types (unconstrained and colour-matched) of model reflectances allow one to assess whether the agreement of model to empirical data is in their detailed form *and* distribution (top), or just in their detailed form (bottom). The figure shows that, to informal inspection, the *PCA* reflectances are a reasonable match in both aspects while the *fourier* ones are not.

**Fig 12:**
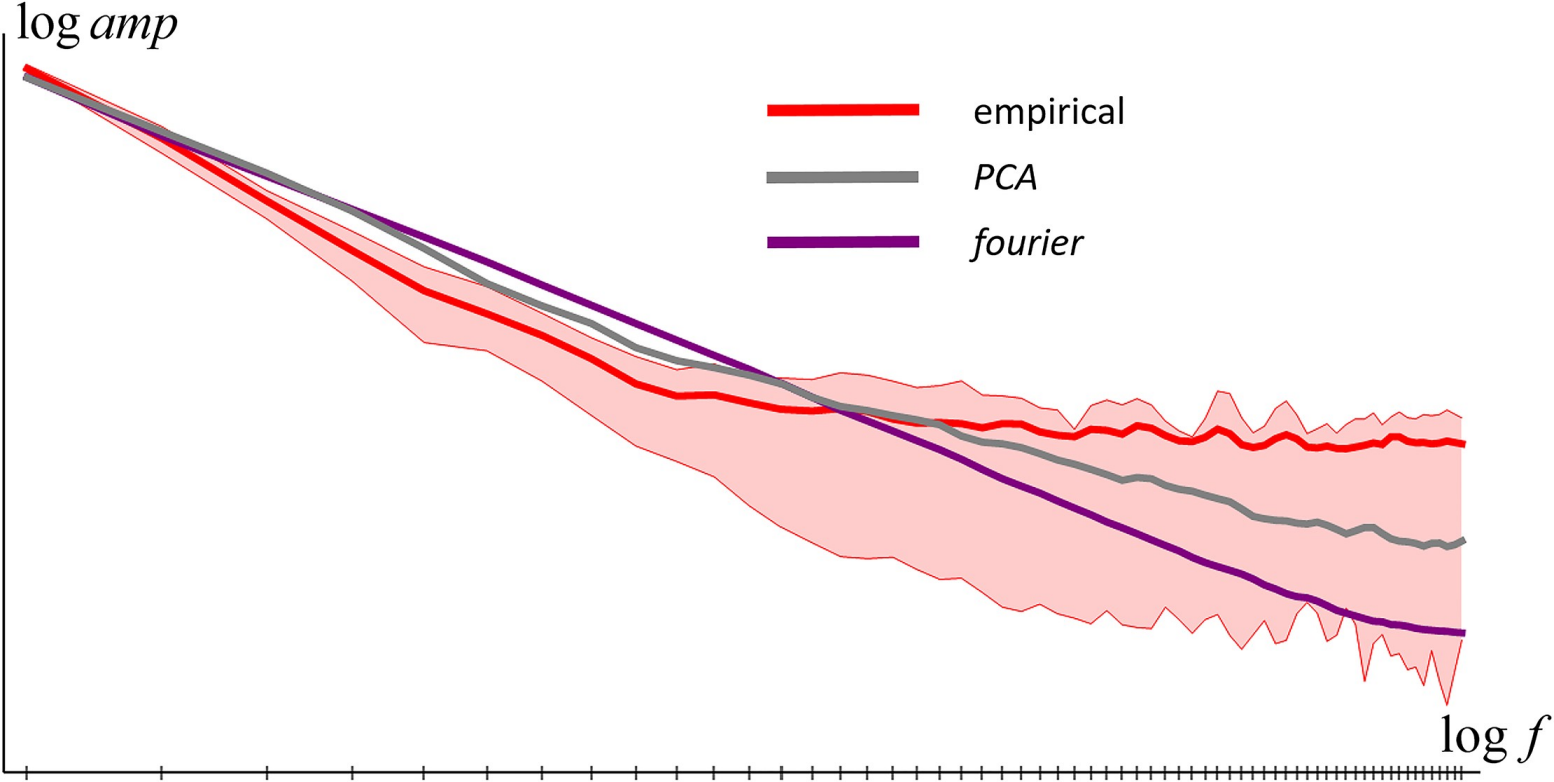
Average fourier amplitude spectra for empirical and synthetic reflectance functions.

In Fig 12 we compare the amplitude spectra of synthetic and empirical reflectance functions. In the low- to mid-frequency range the amplitude fall-off is slower for synthetic functions than empirical, corresponding to their rougher appearance in Fig 11. In the high-frequency range the empirical spectra have higher amplitude than the synthetic–this could easily be improved by adding synthetic measurement noise to the model reflectance functions but we consider this not worth modelling.

**Fig 13:**
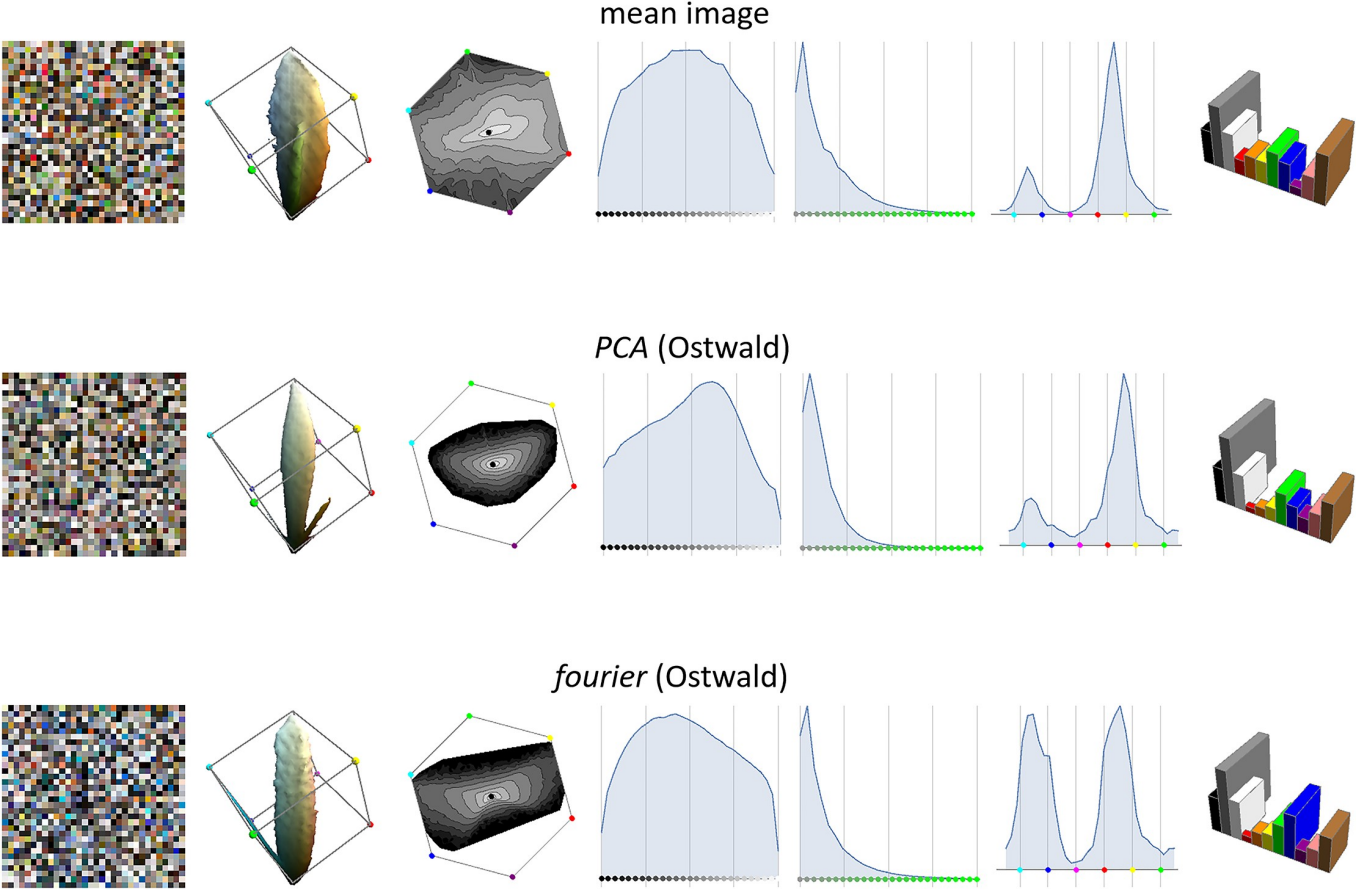
Same layout as Fig 8 but comparing the RGB histogram from the images (top row) to the histograms from two existing models of the distribution of reflectance.

In Fig 13 we compare the RGB histograms that result from the *PCA* and *fourier* models (when using Ostwald formation) to the mean histogram. The *PCA* histogram is quite similar to the mean histogram but its saturations are too low; the *fourier* histogram is very different from the mean histogram, especially in its equal extension in the blue and orange hue directions.

In Table 2 we summarize the realism scores for the *PCA* and *fourier* models. We consider the most important scores to be reflectance realism as measured by the colour-balanced score, and colour realism as measured by the intersection distance (ID) score when using the Ostwald formation model. Based on these we consider *PCA* to be a modest improvement over using the reflectance dataset directly as a model; while *fourier* is slightly worse.

**Table 2:** Realism scores comparing the empirical dataset approach with the two existing models of spectral reflectance. For all metrics lower numbers indicate greater realism. The shaded columns contain the most important realism metrics. The left concerns individual reflectance functions, and the right the distribution of colours arising. The ‘+5’ note for Ostwald-shading reminds of the number of parameters tuned to minimize the ID scores.

		realism scores							
		spectral reflectance		colour histogram					
				uniform-illumination			Ostwald-shading (+5)		
			colour balanced	JSD	EMD	ID	JSD	EMD	ID
**model**	*empirical*	(50%)	(50%)	0.53	4.2%	69%	0.16	2.1%	36%
	*PCA*	74%	55%	0.32	6.8%	53%	0.13	2.2%	30%
	*fourier*	92%	86%	0.49	7.8%	68%	0.14	3.5%	33%

## Improved and new reflectance models

We have developed *PCA+* which aims to be an improved version of *PCA*. The differences are (i) it operates in the latent (*t*) rather than the reflectance domain (*r*), as innovated by [21] (ii) after transformation to the latent domain, empirical reflectances are normalized to zero mean, before computation of the PCA components, (iii) a tunable normally-distributed DC component is used, (iv) a tunable gain factor on the stochastic components is applied. Thus *PCA+* has three tunable parameters which are optimized to minimize the intersection distance between the resulting colour histogram and the mean histogram.

We have developed *fourier+* which aims to be an improved version of *fourier*. The model has a normally-distributed DC term (two parameters), a normally distributed linear slope term (two parameters), and tunable stochastic magnitude. As described in section 2.1, we have independently estimated the power-law coefficient from the reflectance dataset as 2.3, different from the original published estimate of 1.8 [21]. The five tunable parameters are optimized as for *PCA+*.

**Fig 14:**
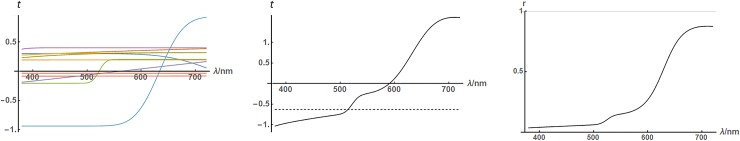
Shows computation of a *sigmoid-sum* reflectance. In this example, the random number of sigmoid components (left) was 10. These are summed and normalized to zero-mean (middle, solid) then added to a random DC component (middle, dotted). The sum is then transformed to the reflectance domain (right).

We have developed a new *sigmoid-sum* model of reflectance functions, inspired by the qualitative appearance of our empirical dataset. The model is explained in Fig 14. It operates in the latent domain. Multiple sigmoid components are summed; each of the form *a*×*erf*((*λ*−*c*)/*σ*), where the error function *erf()* is the version with the range [−1,1], and the amplitude (*a*), centre (*c*) and width (*σ*) are chosen randomly. Each sigmoid is constructed by first choosing its width using a log-normal distribution, then choosing its the centre uniformly in the interval [380−2*σ*, 720+2*σ*] nm; the amplitude is chosen as a normally-distributed random variable independently of the width and centre, and can be positive (increasing sigmoid) or negative (decreasing). The number of sigmoid components in the sum is a random poisson-distributed variable. The sum of sigmoids is normalized to zero-mean before a normally-distributed DC component is added. Finally, the sum is transformed from the latent to the reflectance domain.

The *sigmoid-sum* model has six tunable parameters: mean number of components; mean log-width; mean and sd of the amplitudes; mean and sd of the DC component. These were tuned as for *PCA+* and *fourier+*. The optimal values were: DC component *N*[−0.51, 1.03], *μ* = 10.5 for number of components, widths distributed as *e*^*N*[5.1,1.4]^, and amplitudes distributed as *N*(0.11, 0.36). The distribution of amplitudes is such that 62% of sigmoid components are increasing. The sd of the width distribution was chosen (given the tuned mean) so that the fourier spectra of the resulting reflectance functions had a power-law law fall off with exponent 2.3, as per the empirical data

**Fig 15:**
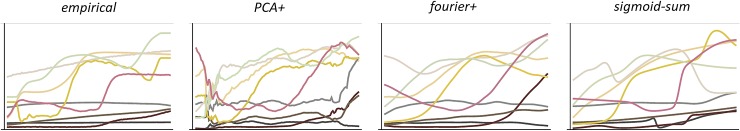
Synthetic reflectance functions (columns 2–4) colour-balanced with the same randomly chosen empirical reflectance functions as in Fig 11 (right).

**Fig 16:**
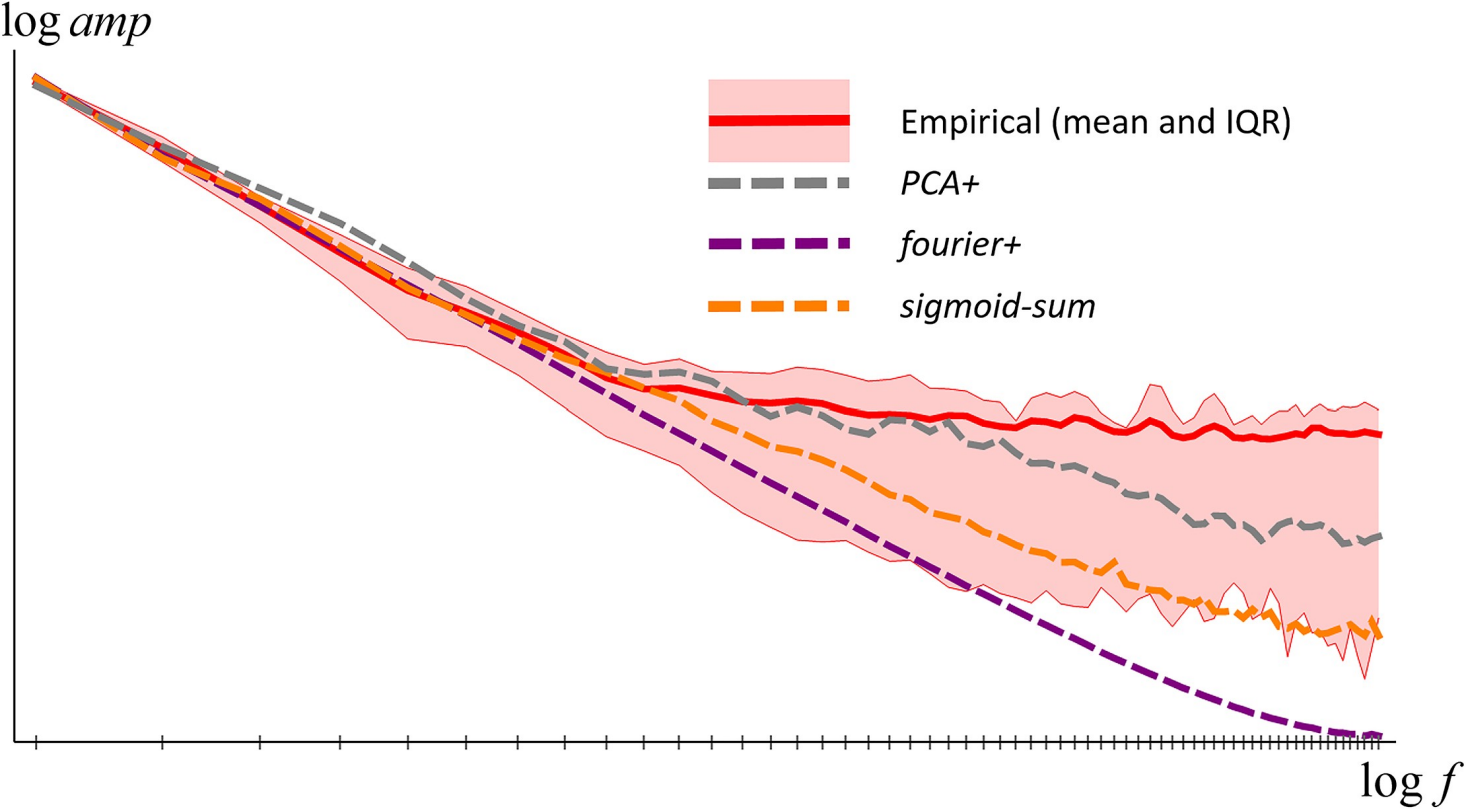
Average fourier amplitude spectra for empirical and synthetic reflectance functions.

**Fig 17:**
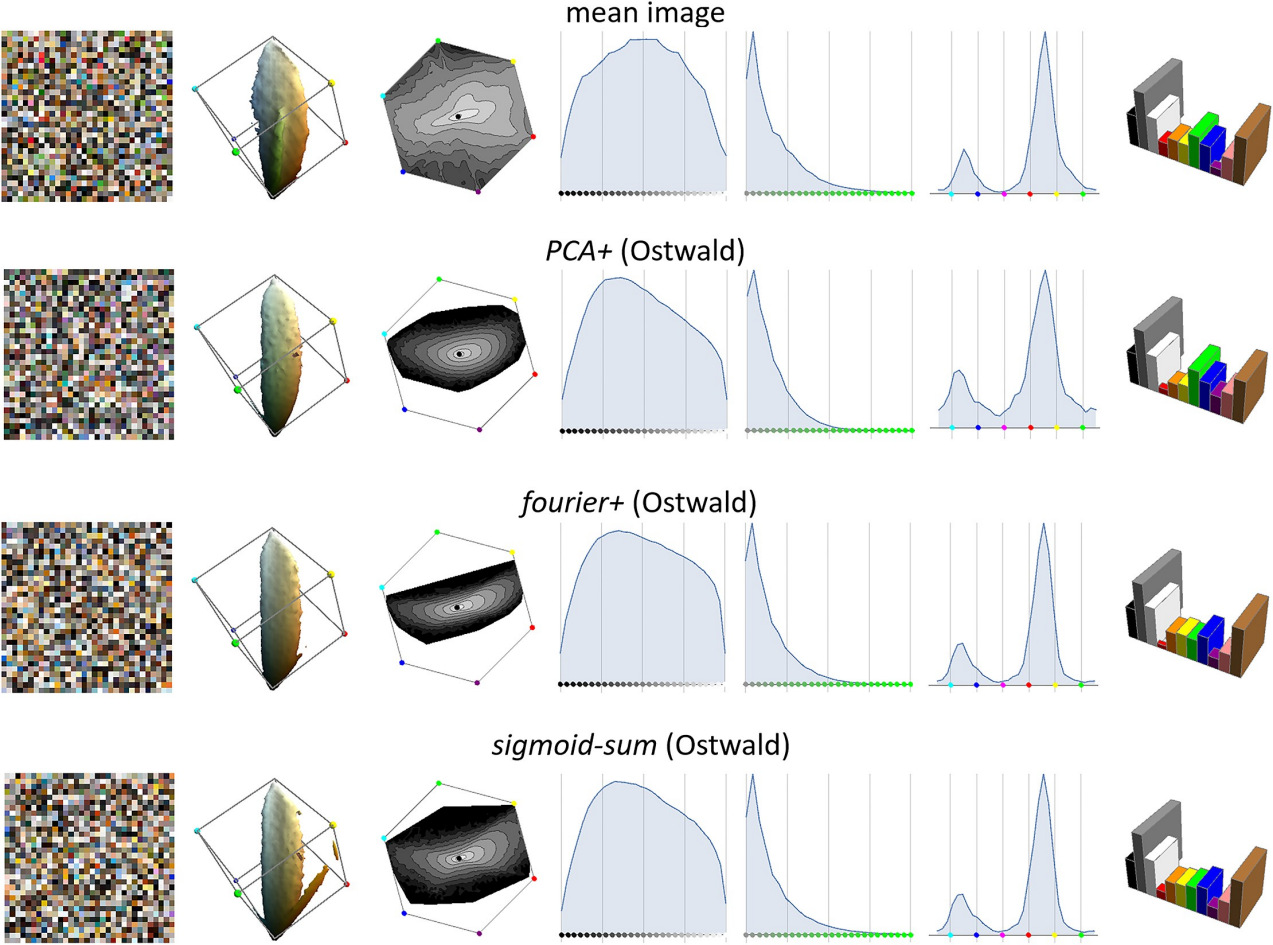
Same layout as Figs 8 & 13.

In Fig 15 we compare reflectance functions for the three new models and in Fig 16 their average fourier amplitude spectra–both figures confirm that all models produce reflectance functions that are more realistic than the previous models. In Fig 17 we show the colour histograms that result from the new models, and all can be seen to be roughly equally similar to the empirical colour histogram.

Figs 15–17 show that the new models are difficult to rank by eye according to their spectral and colour realism. The quantitative assessments of realism presented in Table 3 do allow us to objectively compare them. Observations are:

- *PCA+* produces less realistic reflectances than *PCA*, but slightly more realistic colours.
- *fourier+* is a considerable improvement over *fourier*, and has more realistic reflectances and colours than *PCA+*.
- *sigmoid-sum* is the most realistic model in both key metrics, and in fact has the best scores in all metrics.
- Although *sigmoid-sum* with Ostwald shading has the most tuned parameters (6+5), even with only uniform illumination (6 parameters) it is more realistic than *fourier+* with Ostwald shading (5+5 parameters).

**Table 3:** Realism scores for all models. The shaded columns are the key metrics. Figures in parentheses indicate the number of parameters tuned to minimize ID scores. The best scores in each column are bolded. Final row of table reminds of the difference between top and bottom half-image histograms, given as context.

		realism scores							
		spectral reflectance		colour histogram					
				uniform-illumination			Ostwald (+5)		
			colour balanced	JSD	EMD	ID	JSD	EMD	ID
**model**	*empirical*	(50%)	(50%)	0.53	4.2%	69%	0.16	2.1%	36%
	*PCA*	74%	55%	0.32	6.8%	53%	0.13	2.2%	30%
	*fourier*	92%	86%	0.49	7.8%	68%	0.14	3.5%	33%
	*PCA+ (*3)	79%	60%	0.17	2.0%	38%	0.10	2.1%	28%
	*fourier+* (5)	78%	55%	0.15	1.8%	34%	0.08	1.5%	23%
	*sigmoid-sum* (6)	**68%**	**53%**	**0.07**	**1.5%**	**22%**	**0.06**	**1.0%**	**20%**
image top halves vs. image bottom halves							0.01	3.5%	8%

## Discussion

### 7.1 Key features for realism

Looking across the results we draw conclusions about the key model features needed for realism.

Spectral

- Variation in the mean reflectance as well as variability about that mean is important. *fourier* fails to very realistic (Fig 11) because its functions have the form of excursions from a fixed low reflectance, rather than variations about an intermediate value that itself varies from function to function.
- Reflectance functions need to have the correct frequency content. *PCA* and *fourier* have an excess of mid-frequency power which causes them to be obviously too rough. *PCA+*, *fourier+* and *sigmoid-sum* have correct frequency content and look more realistic as a consequence.
- Correct frequency content alone does not lead to the localized transitions that are visible in some empirical reflectance functions. That requires coordination of phases to align components. Only the *sigmoid-sum* model is able to reproduce these transitions, though possibly to slight excess.
- The explicit modelling of localized step transitions in *sigmoid-sum* gives rise to occasional localized peaks and troughs, in sufficient number to model their rare appearance in empirical functions.

Colour

- As previously observed in [21], the smoothness of empirical reflectance functions leads to orange and blue peaks in the hue marginal. We further note that the orange peak is much higher than the blue (Fig 12), and explain it as due to the greater frequency of positive reflectance slopes than negative. The *PCA* and *PCA+* models capture this tendency in their mean reflectance functions. *fourier* fails to capture the tendency (observe the equal peaks in the hue marginal in Fig 13). This is fixed in *fourier+*, with its explicit modelling of a stochastic linear term which gives a positive slope for the majority of reflectances; and in *sigmoid-sum*, with its preference for individual sigmoid components to be increasing rather than decreasing.
- Purple is notably rare in natural images (Fig 8, top row, hue and Basic Colour marginals). It is more common in the empirical reflectance dataset, possibly because purple samples were included for diversity. *PCA* and *PCA+*, being derived from the empirical reflectances, have too much purple compared to natural images (Figs 13 & 17).

### 7.2. Realism assessed

The new *sigmoid-sum* model scored most highly for both aspect of realism, with the *fourier+* model a close second. The sigmoid-sum realism scores are not perfect though, so it needs to be considered how realistic the model is in absolute terms.

**Fig 18:**
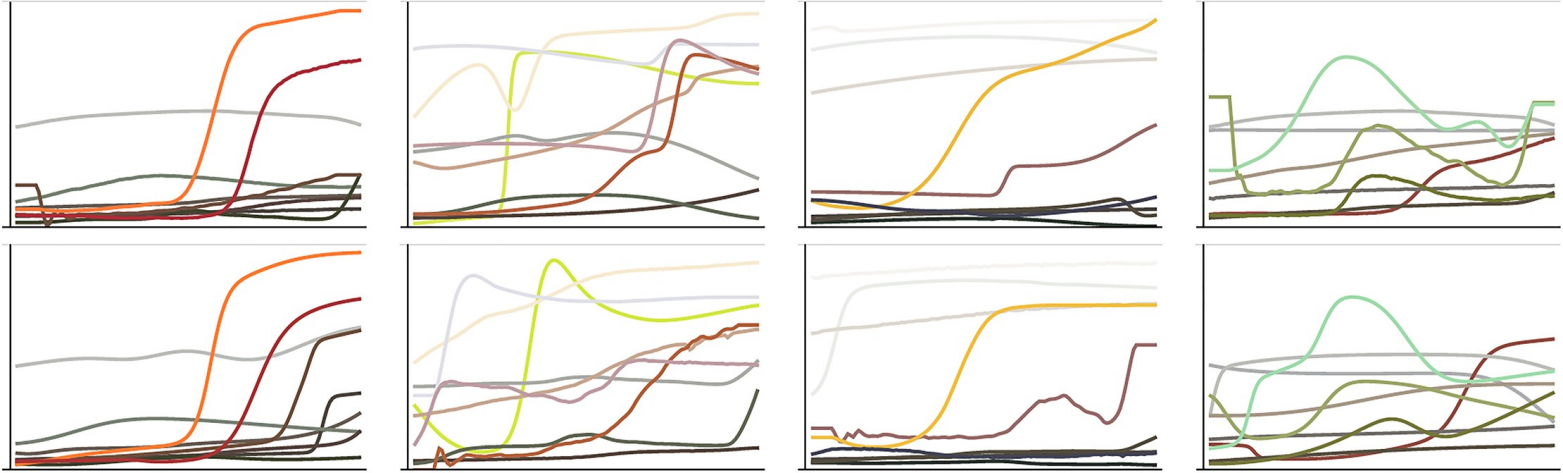
In each column, one panel shows a random selection of reflectance functions (one per collection), the other panel shows colour-balanced *sigmoid-sum* reflectance functions.

First consider the realism of the individual reflectance functions. This was assessed by the performance of a machine learning classifier trained to discriminate between empirical and synthetic functions. The best classifier found was able to do this correctly 53% of the time. This is certainly a low success rate—one would need to observe ~500 trials to become fairly certain that the rate was above chance. However the performance is limited by the ability of the classifier to identify distinguishing features, and while machine learning classifiers are capable of identifying distinguishing features that humans might miss, the same is true vice versa. Ultimately this quantitative assessment of realism should only be considered a supplement to eye measure. In Fig 18 we compare empirical and synthetic functions to allow the reader to make this assessment. The task is certainly possible but not easy, and the most obvious cues are measurement artefacts in the empirical data such as plateaus at short wavelengths or high frequency noise–we could easily simulate these and make the task very difficult. (Empirical functions are in the top row in the outer columns).

**Fig 19:**
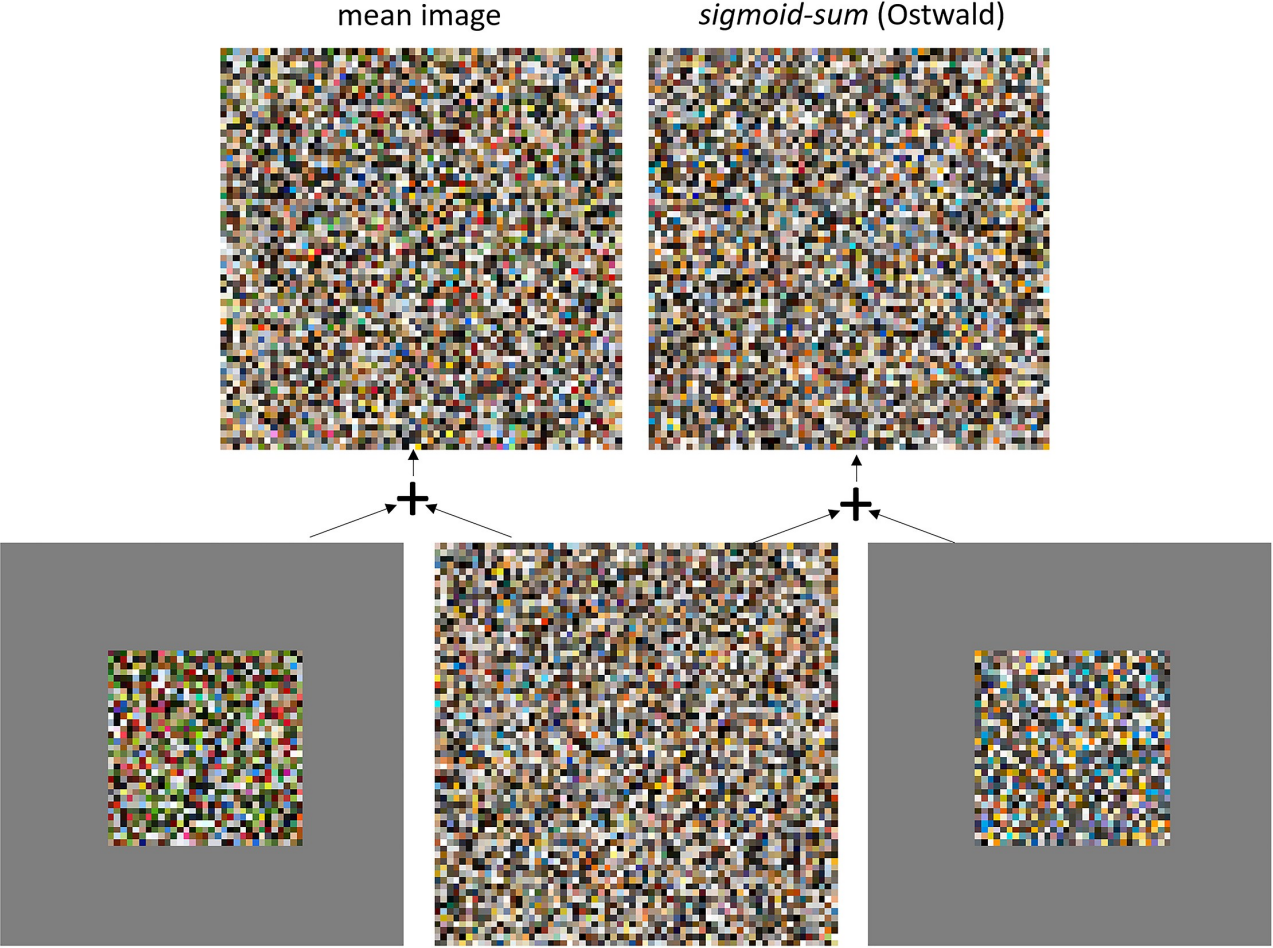
Compares the empirical and model image histograms (top) and shows the breakdown into intersection and excess histograms that give rise to the intersection distance of 20% (bottom). The failure of the model to capture the abundance of vegetation green is apparent in the empirical excess histogram (bottom-left). The models broader range of blues than required to capture sky-blue is apparent in its excess histogram (bottom-right).

Next we consider the realism of the colour histogram for *sigmoid-sum* (Ostwald). Fig 17 visualized this and Table 3 quantified it as an intersection distance of 20%, a Jensen-Shannon Divergence of 0.06 bits, and an Earth Mover’s Distance of 1.0%. In Fig 19 we give a side by side comparison of swatches for the *sigmoid-sum* and mean image histogram. They are visually very similar but some differences can be seen. The difference becomes more apparent in the intersection and excess histograms in the same figure.

The similarities and differences between the model and empirical histograms can be understood from Fig 17 (2^nd^ column). The figure shows that the *sigmoid-sum* model does a good job of accounting for the main leaf-shaped region of high density in the natural image histogram; thus colours from this region can thus be understood as arising from a simple statistical model of reflectance. The *sigmoid-sum* model however fails to model the additional ‘worm’ of green (hypothesized to arise from vegetation) and a ‘fringe’ of light blue (hypothesized to arise from sky) that the mean histogram exhibits. As shown by the excess histograms in Fig 19, it undershoots for the worm and overshoots for the fringe, capturing other blues along with sky blue. No doubt the *sigmoid-sum* model could be made more complex to capture both elements correctly, but we do attempt that here. The model already has five tuned parameters for shading and specularity, and six for the reflectance model. The total count of eleven tuned parameters is not excessive given that a tri-variate normal distribution has nine degrees of freedom, and the mean histogram has a more complicated form than that.

## Summary & conclusions

We have considered models of the distribution of spectral reflectance functions in the natural environment. Assessing them by whether (i) they generate individually realistic functions, and (ii) the distribution of their colours is realistic. For both tests we assess realism by comparison with datasets that we take as representative of the natural environment. We found that a dataset of actual empirical reflectance functions while necessarily individually realistic did not have a distribution of colours that was an excellent match to natural images. A common existing model of reflectance (*PCA*) produced fairly realistic functions and a better distribution of colours than the empirical reflectance dataset, while another existing model (*fourier*) was worse. We were able to slightly improve the *PCA* model (*PCA+*), and significantly improve the *fourier* model (*fourier+*) by incorporating stochastic constant and linear components. While the *fourier+* model can be considered to produce realistic reflectances and colours, we were able to slightly improve on it with a novel *sigmoid-sum* model. We make available for download a large dataset of pre-computed model reflectance functions at doi.org/10.5281/zenodo.3385047 as well as the fuzzy membership functions needed to compute basic colour marginals.

Our model accounts simultaneously for the statistics in two realms of the colour phenomenon–Popper’s World I of physics, where spectral reflectance functions belong–and Popper’s World II of perception, where colours do. Our model raises questions whose answers could extend the theory further down and up. Down into chemistry–why is there a tendency for natural reflectance to increase with wavelength; and up into cognition–are some colours more stable than others under illumination change, and does this correlate with colour categorization as has been suggested [42].

## References

[1] Popper K. Three worlds: Ann Arbor,: University of Michigan.; 1979.

[2] Zeki S, Nash J. Inner vision: An exploration of art and the brain: Oxford university press Oxford; 1999.

[3] Louwerse MM. Embodied relations are encoded in language. Psychonomic Bulletin & Review. 2008;15(4):838–44. 18792513 10.3758/pbr.15.4.838

[4] Fechner G. Elements of psychophysics: Holt, Rinehart & Winston New York; 1965.

[5] Koenderink JJ. Solid Shape: MIT Press; 1990.

[6] Gegenfurtner KR, Sharpe LT. Color vision: From genes to perception: Cambridge University Press; 2001.

[7] Nayar SK, Ikeuchi K, Kanade T. Surface reflection: physical and geometrical perspectives. IEEE Transactions on Pattern Analysis & Machine Intelligence. 1991;(7):611–34.

[8] Born M, Wolf E. Principles of optics: electromagnetic theory of propagation, interference and diffraction of light: Elsevier; 2013.

[9] Oyster CW. The human eye: structure and function: Sinauer Associates; 1999.

[10] Stockman A. Color vision mechanisms: University of Pennsylvania; 2010.

[11] Conway BR, Chatterjee S, Field GD, Horwitz GD, Johnson EN, Koida K, et al Advances in color science: from retina to behavior. The Journal of Neuroscience. 2010;30(45):14955–63. 10.1523/JNEUROSCI.4348-10.2010 21068298 PMC3073527

[12] Chatterjee S, Callaway EM. Parallel colour-opponent pathways to primary visual cortex. Nature. 2003;426(6967):668 10.1038/nature02167 14668866

[13] Mylonas D, MacDonald L, Wuerger S, editors. Towards an online color naming model. Color and Imaging Conference; 2010: Society for Imaging Science and Technology.

[14] Kelley K, Judd D. The ISCC-NBS methods of designating colors and adictionary of color names.[US] Natl. Bur Standards Cir. 1955;553.

[15] Berlin B, Kay P. Basic Color Terms: their Universality and Evolution. Berkeley: University of California Press; 1969.

[16] Griffin LD. Similarity of psychological and physical colour space shown by symmetry analysis. Color Research and Application. 2001;26(2):151–7. WOS:000167092000005.

[17] Foster DH. Does colour constancy exist? Trends in cognitive sciences. 2003;7(10):439–43. 14550490 10.1016/j.tics.2003.08.002

[18] Finlayson GD, Drew MS, Funt BV. Spectral sharpening: sensor transformations for improved color constancy. JOSA A. 1994;11(5):1553–63. 10.1364/josaa.11.001553 8006721

[19] Maloney LT. Evaluation of linear models of surface spectral reflectance with small numbers of parameters. JOSA A. 1986;3(10):1673–83. 10.1364/josaa.3.001673 3772629

[20] Fairman HS, Brill MH. The principal components of reflectances. Color Research & Application. 2004;29(2):104–10.

[21] Koenderink JJ. The prior statistics of object colors. Journal of the Optical Society of America A, Optics, image science, and vision. 2010;27(2):206–17. Epub 2010/02/04. 10.1364/JOSAA.27.000206 . 20126232

[22] Clark RN, Swayze GA, Wise R, Livo KE, Hoefen TM, Kokaly RF, et al USGS digital spectral library splib06a. US Geological Survey Reston, VA; 2007.

[23] Kohonen O, Parkkinen J, Jääskeläinen T. Databases for spectral color science. Color Research & Application. 2006;31(5):381–90.

[24] Webster MA, Mollon J. Adaptation and the color statistics of natural images. Vision research. 1997;37(23):3283–98. 10.1016/s0042-6989(97)00125-9 9425544

[25] Nascimento SM, Ferreira FP, Foster DH. Statistics of spatial cone-excitation ratios in natural scenes. JOSA A. 2002;19(8):1484–90. 10.1364/josaa.19.001484 12152688 PMC1965492

[26] Webster MA, Mizokami Y, Webster SM. Seasonal variations in the color statistics of natural images. Network: Computation in neural systems. 2007;18(3):213–33. 10.1080/09548980701654405 17926193

[27] Koenderink J, van Doorn A. Colors of the Sublunar. i-Perception. 2017;8(5):2041669517733484 10.1177/2041669517733484 28989697 PMC5624368

[28] Hebart MN, Dickter AH, Kidder A, Kwok WY, Corriveau A, Van Wicklin C, et al THINGS: A database of 1,854 object concepts and more than 26,000 naturalistic object images. bioRxiv. 2019:545954. 10.1371/journal.pone.0223792 PMC6793944 31613926

[29] Griffin LD. Optimality of the basic colour categories for classification. Journal of the Royal Society: Interface. 2006;3(6):71–85. 10.1098/rsif.2005.0076 WOS:000235712600007. 16849219 PMC1618485

[30] Breiman L. Random forests. Machine learning. 2001;45(1):5–32.

[31] Zou H, Hastie T. Regularization and variable selection via the elastic net. Journal of the royal statistical society: series B (statistical methodology). 2005;67(2):301–20.

[32] Ohta N, Robertson A. Colorimetry: fundamentals and applications: John Wiley & Sons; 2006.

[33] Stockman A, Sharpe LT. The spectral sensitivities of the middle- and long-wavelength-sensitive cones derived from measurements in observers of known genotype. Vision Research. 2000;40(13):1711–37. 10.1016/s0042-6989(00)00021-3 WOS:000087362500008. 10814758

[34] Anderson M, Motta R, Chandrasekar S, Stokes M, editors. Proposal for a standard default color space for the internet—srgb. Color and imaging conference; 1996: Society for Imaging Science and Technology.

[35] Koenderink JJ. Color Atlas Theory. Journal Of The Optical Society Of America A-Optics Image Science And Vision. 1987;4(7):1314–21. 10.1364/josaa.4.001314 WOS:A1987J043300021.

[36] Foss CE, Nickerson D, Granville WC. Analysis of the Ostwald color system. JOSA. 1944;34(7):361–8.

[37] Griffin LD. Partitive mixing of images: a tool for investigating pictorial perception. Journal of the Optical Society of America A-Optics Image Science and Vision. 1999;16(12):2825–35. WOS:000088951800002.

[38] Coxeter HSM. Introduction to geometry. 1961.

[39] Griffin LD, Mylonas D. Categorical colour geometry. PloS one. 2019;14(5):e0216296 10.1371/journal.pone.0216296 31075109 PMC6510433

[40] Abdi H, Williams LJ. Principal component analysis. Wiley interdisciplinary reviews: computational statistics. 2010;2(4):433–59.

[41] Vrhel MJ, Gershon R, Iwan LS. Measurement and analysis of object reflectance spectra. Color Research & Application. 1994;19(1):4–9.

[42] Olkkonen M, Hansen T, Gegenfurtner KR. Categorical color constancy for simulated surfaces. Journal of vision. 2009;9(12):6–. 10.1167/9.12.6 20053097

